# Group 8 metallocenes as single-source precursors for the synthesis of light-element-stabilized FCC phases under extreme conditions

**DOI:** 10.1080/14686996.2026.2688058

**Published:** 2026-06-19

**Authors:** Ken Niwa, Kenta Nakashima, Yusuke Kimura, Nico Alexander Gaida, Takuya Sasaki, Masashi Hasegawa

**Affiliations:** a Research Center for Crystalline Materials Engineering, Faculty of Engineering, Nagoya University, Nagoya, Japan; b Department of Materials Physics, Faculty of Engineering, Nagoya University Furo-Cho, Nagoya, Japan

**Keywords:** Metallocenes, high pressure synthesis, laser-heated diamond-anvil cell, high pressure *in situ* measurement

## Abstract

Metallocenes are promising single-source precursors for the synthesis of transition-metal carbides and hydrides due to their atomic-scale mixing of metal, carbon, and hydrogen. In this study, we investigated the high-pressure and high-temperature (HP–HT) behavior of group 8 metallocenes, ferrocene [Fe(C_5_H_5_)_2_] and ruthenocene [Ru(C_5_H_5_)_2_], at pressures up to 68 GPa using a laser-heated diamond anvil cell. Optical and Raman spectroscopic observations revealed that both complexes undergo a transition to an opaque state above ~20 GPa, enabling efficient laser coupling. *In situ* synchrotron X-ray diffraction measurements demonstrated that HP–HT treatment of ferrocene and ruthenocene above 30 GPa yielded the formation of the face-centered cubic phase, which indicates that the decomposition of metallocenes under extreme conditions facilitates the stabilization of exotic, light-element-incorporated metallic phases. Our findings highlight the potential of organometallic precursors in exploring the structural diversity of metal-light element systems under ultrahigh pressures.

## IMPACT STATEMENT

This study demonstrates that metallocene decomposition under ultrahigh pressures stabilizes exotic, light-element-incorporated cubic phases, highlighting their potential as single-source precursors for exploring structural diversity in extreme conditions.

## GRAPHICAL ABSTRACT

**Figure:**
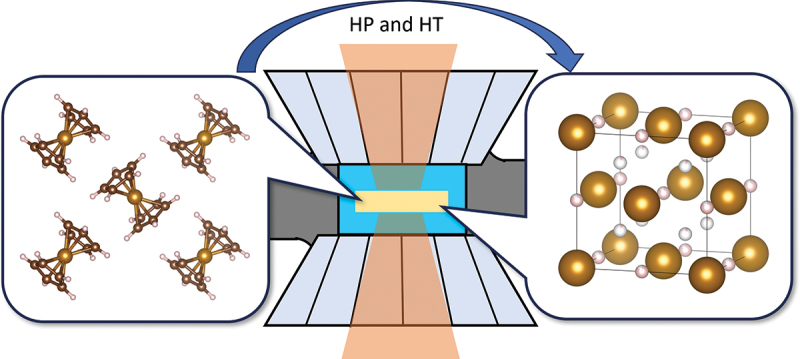

## Introduction

Ferrocene, Fe(C_5_H_5_)_2_, was the first reported metallocene, discovered in 1951 by Kealy and Pauson [1]. Detailed studies subsequently revealed its characteristic sandwich structure, in which an iron center is coordinated between two cyclopentadienyl rings [2–5]. Following this discovery, analogous organometallic complexes of other transition metals, collectively termed metallocenes, were synthesized, stimulating extensive research in organometallic chemistry (e.g. [6,7]). Owing to their unique molecular architectures, metallocenes have attracted considerable interest for their structural responses to external stimuli, including temperature and pressure [8–20]. Their structural stability and phase behavior over wide temperature and pressure ranges have been investigated using X-ray diffraction (XRD) measurements [10–13,15–18,20], spectroscopic techniques [8,19], and DFT calculations [14].

Beyond their fundamental structural significance, metallocenes contain multiple elements at the atomic scale, with relatively high proportions of carbon and hydrogen compared with the transition-metal component. This composition renders them attractive as precursors for the high-pressure synthesis of inorganic compounds, including carbides and hydrides. For example, high-pressure and high-temperature treatments of ferrocene have been conducted using a large-volume press [21–25] or a diamond anvil cell combined with an infrared laser heating system [26]. According to their results, the nanoparticles of carbon-encapsulated iron carbide, iron, and iron carbide were synthesized depending on the conditions. These findings highlight the potential of organometallic complexes as precursors for ultrahigh-pressure synthesis and motivate exploration of broader pressure – temperature regimes and other group 8 metallocenes.

In this study, we employed a diamond anvil cell capable of generating ultrahigh pressures in combination with infrared laser heating to achieve ultrahigh-temperature conditions. This approach enabled the synthesis of inorganic compounds via ultrahigh-pressure /high-temperature treatment of ferrocene and ruthenocene (group 8 metallocenes). To our knowledge, previous synthesis studies using ferrocene were limited to pressures up to approximately 10 GPa and temperatures up to 2200°C [21–25], although room temperature compression experiments had been conducted up to 20 GPa [19]. Furthermore, no high-temperature studies under high pressures have been reported for ruthenocene. Exploration of these metallocenes under previously inaccessible conditions may facilitate the formation of novel carbides, hydrides, or hydrocarbon-containing phases and provide new insights into inorganic material chemistry.

## Experimental

Organometallic complexes of group 8 elements, ferrocene and ruthenocene, were purchased from Fujifilm Wako Pure Chemical Corporation with purities of >98%. The lattice parameters of these starting materials, determined by powder XRD using Cu Kα radiation or synchrotron X-rays, were consistent with previously reported values [27–29].

A diamond anvil cell (DAC) with culet diameters of 350 or 450 µm was used to generate high pressures up to approximately 68 GPa. Stainless steel or rhenium gaskets, initially 250 µm thickness, were preindented, and a sample chamber with a diameter of approximately one-third of the culet diameter was fabricated using a pulsed laser. The organometallic powders were pelletized and loaded into the sample chamber, sandwiched between thin NaCl pellets serving as both a pressure medium and a thermal insulator. Pressure was determined using the ruby fluorescence scale [30]. After compression to the target pressure at room temperature, the samples were heated with a continuous-wave infrared laser irradiation (SPI Lasers UK Limited, *λ* = 1090 nm). Although direct temperature measurements were not performed, thermal radiation ranging from incandescent to white-hot emission indicated that the sample temperature exceeded 2000 K. The laser-heated spot (approximately 10–20 µm) was smaller than the sample size, and the sample position was slowly scanned to achieve uniform heating.

High-pressure and High-temperature-treated products were characterized *in situ* and after recovery using optical microscopy, Raman spectroscopy, and XRD measurements. Raman spectra were obtained with a 473 nm excitation laser (LASOS DPSS laser, Germany), dispersed by an 1800 grooves mm^−1^ grating, and detected with a liquid nitrogen – cooled CCD (Princeton Instruments, U.S.A. PIXIS: 100B_eXcelon). High-pressure *in situ* powder XRD measurements were conducted at beamline BL2S1 of the Aichi Synchrotron Radiation Center [31]. The sample-to-detector distance and X-ray wavelength were calibrated using diffraction patterns from a CeO_2_ standard before the sample measurements. Monochromatic X-rays (λ = 0.75 Å) were incident on the sample through the diamond, and two-dimensional diffraction patterns were recorded on a Quantum 270 CCD detector (ADSC, U.S.A.). To facilitate high-angle diffraction measurements within the DAC, XRD patterns were acquired by tilting the DAC compression axis relative to the incident beam. While the nominal X-ray beam size was set to 50 μm using a collimator, the actual beam profile at the sample position was broader, resulting in occasional signal contamination from the gasket. For specific recovered samples, diffraction patterns were also collected at a longer wavelength (λ = 0.93 Å). The two-dimensional diffraction images were converted to one-dimensional profiles using IPAnalyser [32], and lattice parameters and phase identification were determined using PDIndexer [32]. In total, more than 20 laser-heating experiments (Note: #*n* = run number n) at high pressures were conducted in this study. The products formed through high-pressure and high-temperature treatment are described below based on representative experiments.

## Results and discussion

### Pressure-induced opacity and infrared laser heating

At ambient pressure, both organometallic complexes, ferrocene and ruthenocene, exhibited transparency with yellow and orange characteristic coloration, respectively. With increasing pressure, both organometallic complexes gradually became opaque, reaching a near-complete loss of transparency at pressures above approximately 20 GPa. Raman spectra were collected for both organometallic complexes at selected pressures. Photographs of the sample chamber and the corresponding Raman spectra are presented in Figure S1. The Raman spectra measured at low pressures are in good agreement with previously reported data [8,19,33,34]. Upon compression, the Raman bands systematically shifted toward higher wavenumbers and exhibited progressive broadening. At pressures corresponding to the onset of optical opacity, the Raman intensities decreased drastically, and the Raman signals became nearly undetectable at higher pressures. Although it was difficult to determine whether a distinct structural-phase transition occurred, likely due to nonhydrostatic stress arising from the use of NaCl as the pressure-transmitting medium, the combined optical and spectroscopic observations suggest a pressure-induced transition to a metallic or highly conductive state. The pressure-induced opacity facilitates efficient absorption of near-infrared laser irradiation, enabling laser heating of both ferrocene and ruthenocene at pressures exceeding approximately 16 GPa.

### High-pressure and high-temperature products from ferrocene

**Figure 1.**
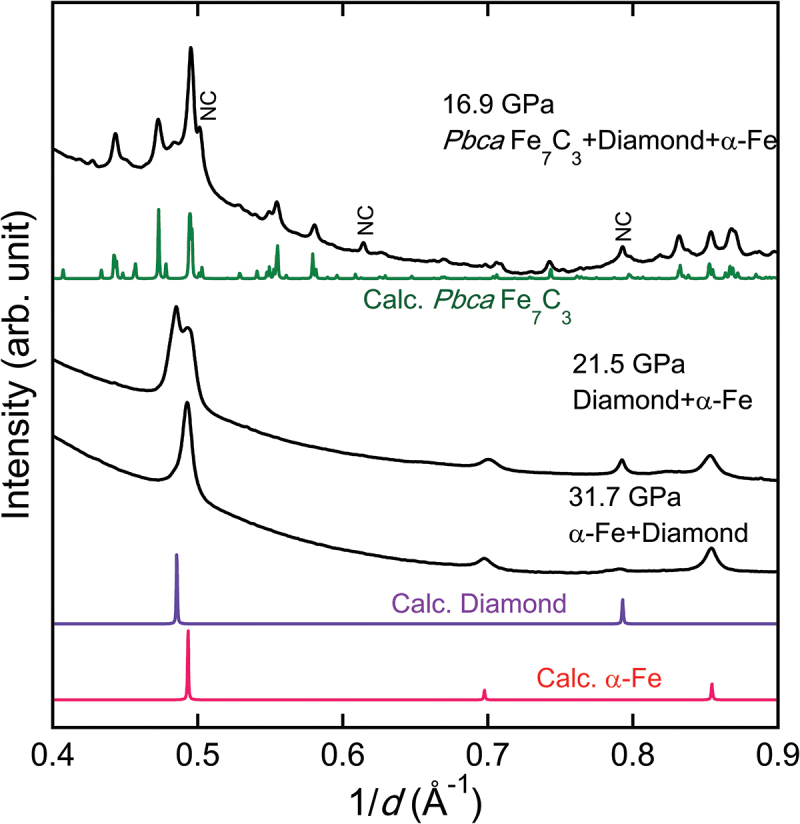
XRD profiles of samples recovered at ambient pressure following laser-heating experiments at 16.9, 21.5, and 31.7 GPa (#10, #46, and #13, respectively). All profiles are plotted against 1/*d* to account for the different X-ray wavelength used in the synchrotron measurements. Synthesized phases for each experiment are indicated below the respective synthesis pressure. Calculated diffraction patterns for *Pbca* Fe_7_C_3_, diamond, and α-Fe are provided for comparison below the experimental profiles. Residual NaCl (denoted as NC), used as the pressure-transmitting medium, was also detected due to incomplete removal.

Figure 1 shows XRD patterns of samples recovered after laser heating of ferrocene under various pressure conditions. The sample recovered after low-temperature laser heating at 16.9 GPa exhibits smooth Debye rings recorded on the detector, and the main peaks of the integrated 1-D profile were assigned to orthorhombic (*Pbca*) Fe_7_C_3_, which are fundamentally consistent with the results of a previous high-pressure study using a laser-heated diamond anvil cell (LHDAC) [26]. Small amount of diamond and α-Fe were also detected. In contrast, the XRD patterns of samples synthesized at higher pressures of 21.5 and 31.7 GPa show markedly simpler diffraction features, consisting primarily of α-Fe and diamond, with no clear evidence of Fe_7_C_3_ formation, although the X-ray intensities relationship between α-Fe and diamond appears to be reversed due to the difference in the phase ratio. Consequently, high-pressure *in situ* XRD measurements are required to elucidate the nature of the phases present immediately after laser heating under high pressure and to monitor their evolution during decompression.

**Figure 2.**
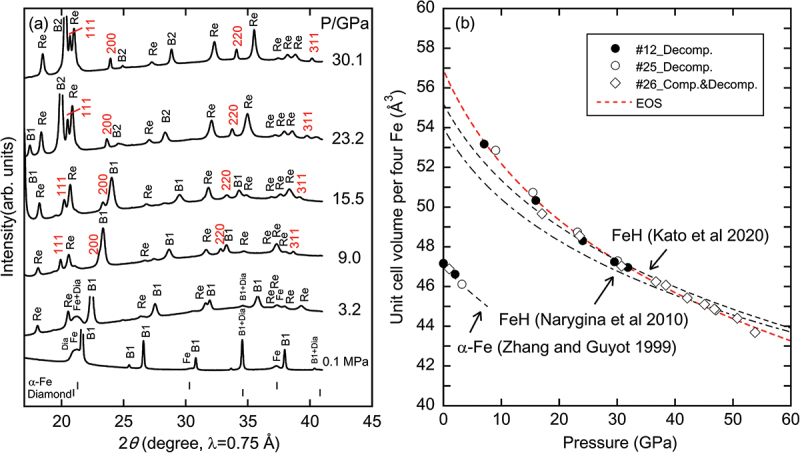
(a) *in situ* high-pressure XRD profiles of the sample after laser heating at 30.1 GPa and during subsequent decompression at room temperature (#25). The labels *hkl*, Fe, and Dia denote diffraction reflections from the cubic phase, bcc-iron (α-Fe), and diamond, respectively. B1 and B2 represent the low- and high-pressure phases of the NaCl pressure-transmitting medium, while Re indicates the rhenium gasket. The ambient-pressure profile was obtained after recovering the sample from the DAC. (b) Pressure – volume (P-V) relationships for the synthesized phase. To facilitate comparison with the compounds having the fcc-alignment of iron, the unit cell volume of α-Fe was normalized to Z = 4. Data from three independent experiments (#12, #25, and #26) are plotted, and the equation of state (EOS) was determined by fitting the combined dataset. The reported EOS data for FeH and α-Fe are included for comparison.

To elucidate the nature of the phases formed immediately after high-pressure laser heating, high pressure *in situ* XRD measurements were performed. Figure S2 shows the XRD patterns collected before and after laser heating at approximately 33 GPa (#12). Several broad diffraction peaks – presumably originating from ferrocene – were observed at low 2θ angles prior to heating; however, these features disappeared upon heating. To further characterize this structural evolution, results from a separate experimental run are described below. Figure 2(a) shows a high-pressure *in situ* XRD pattern collected at room temperature after laser heating at approximately 30 GPa (#25). In addition to reflections from the rhenium gasket, B2-type NaCl (pressure medium), and diamond, several sharp diffraction peaks attributable to a newly formed phase were observed. Upon decompression, these peaks gradually shifted toward lower diffraction angles and disappeared near 3.2 GPa, concomitant with the appearance of diffraction peaks assignable to α-Fe. The newly observed diffraction peaks originate from a single phase and can be indexed to a face-centered cubic (fcc) lattice. Although pure iron is known to crystallize in an fcc structure (γ-Fe) under high-pressure and high-temperature conditions, γ-Fe cannot be quenched to room temperature at high pressure [35–37]. These considerations suggest that the synthesized cubic phase is not pure iron but rather an iron-based compound containing light elements. Pressure – volume (P – V) data obtained for the synthesized fcc phase from three independent experiments (#12, #25, #26) are plotted in Figure 2(b). To facilitate comparison with the compounds having the fcc-alignment of iron, the unit cell volume of α-Fe was normalized to Z = 4. Fitting all P – V data of these experiments using a third-order Birch – Murnaghan equation of state (B-M EOS) yielded zero-pressure bulk moduli (K_0_), its pressure derivative (K_0_^’^), and the ambient pressure unit-cell volume (V_0_) of K_0_ = 85 (22) GPa, K_0_^’^ = 7 (2), and V_0_ = 56.9 (9) Å^3^, respectively. The uncertainty might be due to the data scattering derived from the unstable fcc phase under low-pressure conditions, but the calculated values are fundamentally consistent with recently reported values for FeH [38,39]. Furthermore, the decompression behaviors in which the fcc phase decomposed into α-Fe, are also consistent with the result of previously reported FeH [40] and α-Fe [41]. The decomposition phases of laser-heated ferrocene, excluding iron-based compounds, were also investigated using Raman spectroscopy (#80, Figure S3). Given the chemical composition of ferrocene, various light-element materials, such as H_2_, C, and C_x_H_y_, were expected upon decomposition. After heating at approximately 30 GPa, no distinct peaks were observed in the 4000–4500 cm^−1^ range, which corresponds to the vibrational stretching modes of molecular hydrogen [42]. However, a weak, broad Raman feature was locally observed around 3100 cm^−1^. This feature was consistently observed across multiple experimental runs, indicating the reproducibility of our findings. The peak intensity increased during decompression but disappeared upon opening the DAC sample chamber. Based on the precursor’s chemical composition and previously reported Raman profiles, this peak is likely attributed to methane (CH_4_) [43,44]; its absence in the XRD patterns is presumably due to its low scattering factor and low abundance. Additionally, diamond synthesis was confirmed in several other runs. While these results indicate the formation of light-element compounds as decomposition products, precise temperature control in LHDAC experiments remains inherently challenging, potentially yielding various alternative phases depending on specific conditions. Therefore, further detailed investigations are indispensable for a comprehensive understanding of the decomposition mechanism under extreme conditions.

Further experiments at higher pressure (#26) were conducted to examine the pressure dependence of the synthesized cubic phases. When ferrocene was laser-heated at approximately 30 GPa and subsequently compressed to approximately 50 GPa at room temperature, the fcc phase remained stable under compression, as confirmed by high-pressure *in situ* XRD measurements (lower profile of Figure S4 and Figure 2(b)). In contrast, laser heating at approximately 50 GPa resulted in the emergence of new diffraction peaks that can be indexed to a double hexagonal close-packed (dhcp) structure (upper profile of Figure S4). Recent high-pressure and high-temperature study of iron hydrides have shown that fcc-FeH remains stable below the melting curve at pressures exceeding 100 GPa, whereas dhcp-FeH is the thermodynamically stable low-temperature phase (<1200 K at 50–60 GPa) [39]. The present synthesized phase after heating at 50 GPa is consistent with the previously reported dhcp-FeH phase [39]. Pressure – volume (P – V) data obtained for the dhcp phases are shown in Figure S4. Similarly as for the fcc phase, K_0_ = 141 (6) GPa (K_0_^’^ = 4 fixed), and V_0_ = 55.3 (3) Å^3^ were obtained by fitting the P-V data to the B-M EOS and are in good agreement with previously reported values for dhcp-FeH (K_0_ = 131.1 GPa, K_0_^’^ = 4.83, and V_0_ = 55.6 Å^3^) [45]. Taken together, these results indicate that the laser heating of ferrocene below 20 GPa favors the formation of Fe_7_C_3_ and diamond, whereas at higher pressures (30–50 GPa), iron hydride-related phases are preferentially formed. Nevertheless, the potential incorporation of carbon into the synthesized iron-based compounds (e.g. FeC_x_H_y_) cannot be ruled out and remains a critical subject for future studies.

### High-pressure and high-temperature products from ruthenocene

**Figure 3.**
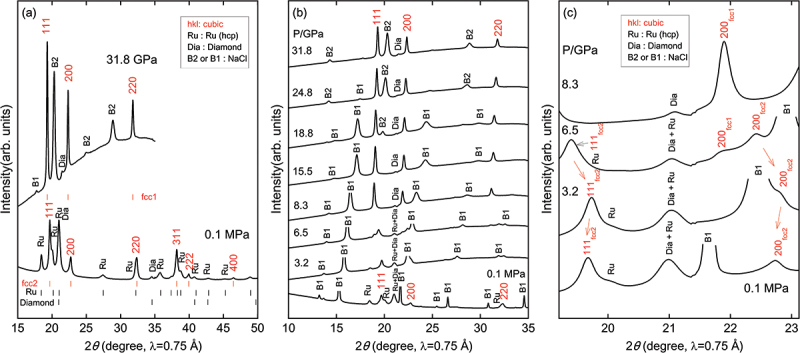
(a) XRD profiles collected at 31.8 GPa and at ambient pressure after decompression (#31). The labels *hkl* denote reflections from the cubic phases, designated as fcc1 and fcc2. Following pressure release, the sample was recovered from the DAC, and washed with distilled water to remove the NaCl pressure-transmitting medium. (b) *in situ* XRD profiles recorded during decompression. Diffraction reflections from the cubic phase are indexed with hkl. Unlabelled peaks are assigned to the B1 and B2 phases of NaCl, diamond, and hcp-ruthenium. (c) Magnified XRD profiles in the 2*θ* range of 19–23°. The subscripts fcc1 and fcc2 represent the fcc phases observed immediately after high-pressure heating and during the decompression process, respectively.

High-pressure and high-temperature experiments on ruthenocene were performed over a pressure range from 20 to 68 GPa. Similar to ferrocene, ruthenocene became opaque above approximately 20 GPa (Figure S1), enabling efficient near-infrared laser heating. Figure 3(a) shows the high-pressure *in situ* XRD pattern collected at room temperature after laser heating at 30 GPa. In addition to reflections from NaCl and diamond, several diffraction peaks were observed that cannot be assigned to pure ruthenium in its hexagonal close-packed (hcp) structure. Indexing of these peaks indicates that they can be described by an fcc lattice, similar to the behavior observed in the ferrocene experiments. XRD patterns collected during decompression are shown in Figure 3(b). The diffraction peaks associated with the fcc phase shifted continuously toward lower angles as pressure decreased. At approximately 6.5 GPa, peak splitting was observed, resulting in the emergence of two distinct cubic phases, designated fcc1 and fcc2, along with diffraction peaks corresponding to an hcp phase (Figure 3(c)). Upon further decompression, the fcc1 phase disappeared, whereas the fcc2 and hcp phases were retained and recovered to ambient pressure (Figure 3(c)). The lattice constants of the recovered hcp phase (*a* = 2.699–2.7039 Å, *c* = 4.290–4.301 Å, variation due to the runs) are almost identical to the literature values for Ru (*a* = 2.705 Å, *c* = 4.280 Å) [46].

**Figure 4.**
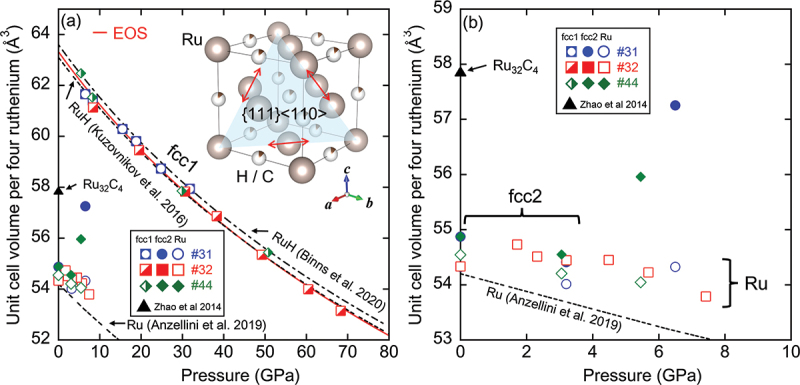
Pressure – volume relationships of products obtained from the high-pressure heating of ruthenocene. Data from three independent experiments (#31, #32, and #44) are plotted, along with the equation of state (EOS) derived from the combined dataset. Figure 4(a) shows the P – V relationships for all data, while (b) displays the region below 10 GPa. The half-filled, filled, and open symbols correspond to the data for fcc1, fcc2, and hcp-Ru, respectively. For comparison, the reported EOS for RuH, pure Ru, and the fcc-Ru_32_C_4_ phase synthesized via shock wave experiments are also displayed. The crystal structure of the synthesized fcc phase, modelled after a NaCl-type structure, is shown with the {111} plane and <110>slip direction indicated. In this model, light elements (hydrogen or carbon) occupy the octahedral interstitial sites.

Ru–C, Ru–H, and Ru–C–H compounds are considered as candidate materials for the cubic phase. We first compare the high-pressure behavior and stability of the cubic phase with those of the previously reported RuH, which crystallizes in a NaCl-type structure [47–49]. Figure 4 shows the pressure dependence of the unit-cell volume for the fcc1, fcc2, and hcp phases during decompression. Fitting P – V data of the fcc1 phase for three independent runs to a second-order Birch – Murnaghan equation of state (BM-EOS) yields the following K_0_ = 280 (6) GPa, K_0_’ = 4 (fixed), and V_0_ = 63.3 (1) Å^3^. These values are in good agreement with the previously reported parameters for RuH (K_0_ = 290 (30) GPa, K_0_^’^ = 4 (fixed), V_0_ = 63.1 (7) Å^3^ [47]; K_0_ = 272 (27) GPa, K_0_^’^ = 4.1 (10), V_0_ = 63.6 (4) Å^3^ [49]). Previously reported RuH was found to decompose completely into elemental ruthenium and molecular hydrogen during decompression from 8.1 to 6.2 GPa [47]. Furthermore, Binns et al. reported that heating RuH in H_2_ at temperatures above 1500 K and pressures of approximately 54 GPa resulted in the formation of Ru_3_H_8_ [49]. In contrast, in the present study, the fcc1 phase persisted upon recompression and reheating above approximately 50 GPa, and no diffraction peaks corresponding to Ru_3_H_8_ were observed. In addition, as described earlier, the cubic phase was retained, and the recovered hcp phase exhibited almost identical ambient-pressure lattice parameters to, but a higher bulk modulus than, those reported for pure hcp Ru [46,50]. This may indicate partial carbon incorporation into the ruthenium lattice (see Figure 4). Taken together, these results suggest that the cubic phase synthesized in this study adopts an fcc ruthenium arrangement similar to that of fcc-RuH [47,49] and may contain some carbon, particularly within the fcc2 phase.

### Formation of cubic phases and role of light elements in stabilizing fcc framework

The present results demonstrate that high-pressure and high-temperature treatment of group 8 metallocenes above approximately 20 GPa consistently leads to the formation of metal-based fcc phases. The emergence of fcc phases in both systems suggests a common formation pathway involving close-packed metal frameworks stabilized by light elements released from the organometallic precursors. In the ferrocene experiments, the fcc phase appears above approximately 20 GPa and decomposes into α-Fe upon decompression, a behavior consistent with that of previously reported iron hydrides [40]. Unlike the case of ferrocene, the experimental results for ruthenocene are not straightforward. Although RuH is known to crystallize in an fcc structure, it decomposes completely during decompression [47,49]. In contrast, the cubic phase synthesized from ruthenocene partially survives decompression and can be recovered to ambient pressure, indicating an additional stabilization mechanism beyond hydrogen incorporation alone.

The contrasting decompression behaviors of the iron- and ruthenium-based fcc phases highlight the importance of the host metal lattice. At ambient pressure, iron is stable in the body-centered cubic (bcc, α-Fe) structure, which involves a substantial rearrangement of atomic coordination relative to the fcc lattice. In contrast, ruthenium adopts the hexagonal close-packed (hcp) structure over a wide range of temperatures and pressures [46,50–52]. The structural relationship between the fcc and hcp lattices is governed by differences in the stacking sequence of close-packed atomic planes; accordingly, the fcc-to-hcp transformation proceeds via a martensitic mechanism involving shear deformation along the ⟨110⟩ direction on the {111} planes (insertion figure of Figure 4). This transformation pathway suggests that the fcc phase could, in principle, be retained if slip along the close-packed planes is inhibited. Under high-pressure and high-temperature conditions, hydrogen is expected to preferentially occupy interstitial sites within the fcc metal framework, while carbon may also be incorporated through decomposition of ruthenocene. During decompression, hydrogen is released more readily, whereas carbon may remain within the lattice and contribute to the stabilization of the fcc2 phase.

### Implications for materials synthesis using organometallic precursors

Overall, this study demonstrates that organometallic complexes can serve as versatile single-source precursors for the synthesis of novel light-element-containing cubic phases under extreme pressure – temperature conditions. The intrinsic presence of hydrogen and carbon at the atomic scale facilitates reaction pathways that are difficult to access via conventional solid – solid reactions, offering a general strategy for stabilizing metastable metallic frameworks. As the remaining elemental component alongside ruthenium and hydrogen, carbon likely contributes to suppressing the fcc-to-hcp transition at low pressures. This interpretation is supported by Zhao et al., who successfully synthesized the carbon-deficient fcc phase, Ru_32_C_4_, via shock compression [53]. In their study, samples were rapidly quenched from ultrahigh-pressure and ultrahigh-temperature conditions to ambient conditions on extremely short timescales, resulting in nonequilibrium recovery. Such rapid quenching likely suppressed long-range carbon diffusion, enabling the retention of the carbon-containing fcc phase. In contrast, static synthesis studies using laser-heated diamond anvil cells to react ruthenium and carbon directly have reported the formation of a more carbon-rich phase [54]. We also conducted separate high-pressure and high-temperature synthesis experiments using starting materials prepared from metallic ruthenium and carbon mixtures via arc melting followed by rapid quenching (Figure S5). However, no carbide phases were obtained at pressures above 20 GP, and the nature of this discrepancy with the previous study remains unclear, which might be derived from the difference in the experimental setup.

The present results suggest that diffusion-driven solid-state reactions at the Ru–C interface are kinetically hindered even under high-pressure conditions. In our static high-pressure experiments, the fcc phase was synthesized using organometallic complexes that intrinsically contain large amounts of hydrogen and carbon. Under high-pressure and high-temperature conditions, hydrogen preferentially occupies interstitial sites within the fcc lattice, while carbon atoms, liberated upon the decomposition of the complexes, may partially occupy the same sites. During decompression, hydrogen is released from the lattice, whereas the remaining carbon likely stabilizes the fcc structure. Notably, the lattice parameter of the cubic phase of the ruthenium system recovered at ambient pressure (*a* = 3.800 Å, *V* = 54.87 Å^3^) is smaller than that reported for Ru_3__2_C_4_ (*a* = 3.868 Å, *V* = 57.87 Å^3^) [53], indicating a lower carbon concentration, probably due to the coexistence of hydrogen. On the other hand, this value is slightly larger than the volume (54.2 Å^3^) calculated under the assumption that the fcc-Ru phase maintains the same volume per atom as the hcp phase (approximately 13.55 Å^3^ atom^−1^). According to the chemical synthesis study by Kusada et al., both fcc and hcp ruthenium (Ru) nanoparticles (2.0–5.5 nm) can be selectively synthesized via chemical reduction using specific metal precursors [55]. Their results demonstrated that the fcc phase is stabilized at ambient pressure through the nanosize effect, specifically by being bounded by {110} facets, which leads to distinct catalytic properties [55]. The fcc phase synthesized in this study was stabilized by the incorporation of a small amount of carbon, maintaining high crystallinity and large crystallite sizes despite the high-temperature and high-pressure conditions. This study underscores that high-pressure synthesis utilizing organometallic precursors containing multiple light elements at the atomic level is a highly effective strategy for developing functional materials that are otherwise inaccessible through conventional solid-state diffusion.

## Summary

Organometallic complexes were subjected to high-pressure and high-temperature (HP–HT) treatment at approximately 16–68 GPa using a laser-heated diamond anvil cell (LHDAC). The resulting phases were characterized via Raman spectroscopy, *in situ* and *ex situ* XRD. For ferrocene, laser heating below 20 GPa yielded the previously reported Fe-C compound. In contrast, heating at higher pressures of 30 GPa led to the formation of a face-centered cubic (fcc) phase, which was found to decompose completely upon decompression. Similarly, ruthenocene treated around 30 GPa produced an fcc-structured compound (fcc1), which subsequently decomposed into an hcp phase and another fcc phase (fcc2) with a smaller unit-cell volume than fcc1 during decompression. These synthesized phases are considered to consist of an fcc metal framework with interstitial hydrogen and carbon atoms. This study demonstrates that high-pressure and high-temperature treatment of organometallic precursors, combining high-pressure *in-situ* measurements is a highly effective strategy for developing functional materials that are otherwise inaccessible through conventional solid-state diffusion process.
